# Unrevealing the Epigenetic Landscape: SOX-2 and OCT-4 methylation in acute myeloid leukemia and myelodysplastic neoplasm

**DOI:** 10.15190/d.2026.8

**Published:** 2026-05-31

**Authors:** Damini Singh, Manish Kumar, Geeta Yadav, Uma Shankar Singh, Shantanu Prakash, Rashmi Kushwaha, Mili Jain, Shailendra Prasad Verma

**Affiliations:** ^1^Department of Pathology, Autonomous State Medical College, Hardoi, India; ^2^Department of Clinical Hematology, King George's Medical University, Lucknow, India; ^3^Department of Pathology, King George's Medical University, Lucknow, India; ^4^Precision Medicine Unit, Department of Critical Care Medicine, King George's Medical University, Lucknow, UP, India

**Keywords:** Acute myeloid leukemia, AML, myelodysplastic neoplasm, MDS, SOX-2, OCT-4, methylation.

## Abstract

Epigenetic modifications, particularly aberrant DNA methylation, play an important role in the pathogenesis of both solid and hematologic malignancies. Global hypomethylation and promoter hypermethylation can silence numerous tumor suppressor genes identified in acute myeloid leukemia (AML) and myelodysplastic neoplasm (MDS). Among stemness-associated transcription factors, OCT-4 and SOX-2 are key regulators of pluripotency and stem cell maintenance and have been implicated in cancer stem cell biology and tumor progression. Although their abnormal expression and epigenetic regulation have been reported in various malignancies, limited data are available regarding their promoter methylation status specifically in AML and MDS. Therefore, this study aimed to evaluate the methylation patterns of the transcription factors, specifically OCT-4 and SOX-2, in patients with AML and MDS. The study involved 84 newly diagnosed AML and MDS patients and 16 age, and sex-matched healthy controls. DNA was extracted from blood or bone marrow using a QIAGEN RDNA extraction kit. The SOX-2 and OCT-4 genes were studied using PCR-specific primers for methylated and unmethylated targets. SOX-2 gene methylation was observed in a significantly higher proportion of AML (n=48/77) and MDS (n=4/7) as compared to Controls (n=3/16) (p<0.001). OCT-4 methylation was also observed in a significantly higher proportion of AML (n=44/77) as compared to MDS (n=3/7) and Controls (n=1/16) (p<0.001). Only a few cases in the AML group had both SOX-2 and OCT-4 gene methylation (n=25/77), compared with MDS (n=0/7) and Controls (n=0/16) (p<0.007). This study indicates that SOX-2 and OCT-4 gene methylation is significantly more prevalent in AML and MDS patients than healthy controls, suggesting their potential involvement in leukemogenesis. These findings highlight the potential role of methylation of stemness-associated transcription factors as a biomarker for disease characterization and identification of epigenetic therapeutic targets in AML and MDS.

## INTRODUCTION

Acute myeloid leukemia (AML) is a heterogeneous disease and a result of abnormal myeloid blast accumulation in the bone marrow that, eventually, contributes to bone marrow failure^1,2^. The global occurrence of acute leukemia is 0.01%, and death is ~8/100000 per year^3^. The incidence of AML is rising globally from 79372 in 1990 to 144654 in 2021^4^. Myelodysplastic neoplasm (MDS) is a heterogeneous condition of clonal hematopoietic disorders characterized by ineffective erythropoiesis, dysplastic features, chromosomal abnormalities, and increased risk of AML progression. The global incidence of MDS ranges from .06 to 0.26/100000^5^. The progression of MDS to AML is an example of the multistep theory of carcinogenesis^6^.

Epigenetics is the mechanism that alters gene expression without affecting the genetic sequence^7,8^. They commonly include alterations in chromatin structure or interference with RNA transcripts, DNA methylation, histone modifications, nucleosome remodeling, and noncoding RNAs^9^. Alterations in DNA methylation are predominantly found in solid tumors, including hematological malignancies. Global hypomethylation and promoter hypermethylation can silence tumor suppressor genes, as described in AML and MDS^10,11^.

It has been observed that the Leukemic cell population represses embryonic cell markers SOX-2 and OCT-3/4, which play a crucial role in suppressing genes involved in cell differentiation and in self-renewal. SOX (sex-determining region Y-Box) is a family of proteins with a host of transcriptional factors crucial in embryogenesis. SOX-2, SOX-4, SOX-9, and SOX-18 were oncogenes promoting cancer growth. SOX-6, SOX-7, and SOX-17 are tumor suppressor genes, whereas SOX-10 functions as both an oncogene and a tumor suppressor^12^. The Sox family genes have been identified as regulators of various developmental processes, including the maintenance of pluripotency in embryonic stem cells, chondrogenesis, testis development, and brain and neural tissue development. The SOX gene family shows differential expression in AML, either being silent in AML, suggesting a potential tumor-suppressor role, or being enhanced, indicating a potential oncogenic role^13^. Octamer 4/OCT-4 (OCT-3, OCT-3/4, and POU5F1) is a POU family member expressed in the germ line and pre-gastrulation embryo^14,15^. OCT-4 is essential in maintaining the self-renewal and totipotency of embryonic stem cells^16,17^. OCT-4, SOX-2, Klf-4, c-Myc, and Nanog can help reprogram somatic cells, producing induced pluripotent stem (iPS) cells^18-21^. Various studies highlight the significance of SOX2 gene methylation in solid tumors but very few in hematological malignancies.

Regarding MDS and AML, the current challenge is selecting an appropriate combination of treatment modalities. Several DNA methylation events affect gene expression, but their potential as biomarkers for early diagnosis, stratification, and prediction of treatment response has yet to be thoroughly evaluated, as changes in DNA methylation are early manifestations and may also serve as therapeutic targets. Because DNA methylation is reversible, it is a promising therapeutic target. The effects of hypomethylating agents on the treatment of patients with myelodysplastic neoplasms (MDS) and AML have been assessed^22,23^.

Compared with several other stemness-related genes, OCT-4 and SOX-2 were selected for their established roles in regulating stem cell plasticity and epigenetic alterations in cancer biology^24^. However, studies evaluating their promoter methylation status specifically in AML and MDS are limited. With the availability of hypomethylating agents for myeloid malignancies, knowledge of methylation status would help guide response prediction. Therefore, this study aimed to evaluate the methylation patterns of the transcription factors OCT-4 and SOX-2 in patients with AML and MDS.

## MATERIALS AND METHODS

This was a prospective observational study (between July 2019 and December 2020) conducted in the Department of Pathology, in collaboration with the Department of Clinical Hematology at King George’s Medical University, Lucknow, India, after being approved by the Institutional Ethical Committee (Ref-Code: 97th ECM II B-Thesis/P36 dated 19.07.2019). Morphologically and immunophenotypically diagnosed cases of AML (n=77) and MDS (n=7) were included in the study. AML secondary to therapy or other malignancies were excluded from the study. Therapy-related AML patients were excluded to rule out possible drug effects. Blood samples from 16 age-matched healthy controls were also collected. Written informed consent was taken from all the participants. The research was conducted in accordance with the national and institutional guidelines for human participation and accordance with the principles of the Helsinki Declaration.

Two millilitres (ml) of bone marrow or venous blood in an Ethylenediamine tetraacetic acid (EDTA) vial were collected from newly diagnosed cases of AML and MDS for the methylation study. Bone marrow was collected from the majority (69/77) of AML patients, as marrow samples had already been drawn for other investigations, including flow cytometry and cytogenetic studies. In 7 patients, marrow samples were unavailable (e.g., dry tap-4, insufficient sample-2, patient refused-1). For MDS, peripheral blood samples were used for methylation studies due to the unavailability of a bone marrow sample.

### DNA methylation analysis protocol

Sample Collection and DNA Extraction:

Venous blood or bone marrow samples were collected and processed using a QIAGEN ^R^DNA extraction kit, ensuring the retrieval of high-quality genomic DNA for subsequent analysis. The extracted DNA underwent bisulfite treatment, which converts unmethylated cytosines to uracil while preserving methylated cytosines. The methylation-specific PCR was performed as described by Zhang et al^25^. This treatment facilitates the differentiation between methylated and unmethylated DNA regions. Specific primers targeting the methylated and unmethylated regions of the SOX-2 and OCT-4 genes were synthesized (**Table 1**).

**Table 1:** Primer sequences and PCR conditions used for methylation-specific PCR analysis of SOX-2 and OCT-4 genes M: methylated, UM: unmethylated; FP: Forward Primer, RP: Reverse Primer; t`: annealing temperature

Gene	Primers	FP/RP	t	Primers 5’-3’	Size
SOX-2	M-MSP	FP	55	TTAATAAGAGAG TGGAAGGAAATTTAGA	214
		RP		AAATAAAAACTC AAAAATCTTACCCG	
	U-MSP	FP	55	TAATAAGAGAGT GGAAGGAAATTTAGAT	212
		RP		ATAAAAACTCAA AAATCTTACCCACC	
OCT-4	M-MSP	FP	55	ATAAAAATAGGA AGGAGTTTTTCGC	200
		RP		CTATCAAACAAA TACAACAACGTCG	
	U-MSP	FP	53	AAAATAGGAAG GAGTTTTTTGTGA	196
		RP		ATCAAACAAAT ACAACAACATCAAC	

The polymerase chain reaction (PCR) mixture contained 10 pm of each primer, 2.5 µl of 10X PCR buffer, 0.8 µl of 10 mM dNTP mix, 1 U of Taq polymerase, 5 µl of treated DNA, and nuclease-free water to adjust the total volume to 25 µl. The amplifications were carried out on a thermal cycler system with the following conditions: 95°C for 10 min, followed by 35 cycles at 95°C for 15 s, 55°C for 1 min, and 72°C for 1 min. After amplification, PCR products were run on an agarose gel and visualized after staining with ethidium bromide.

#### Statistical analysis

The statistical analysis and correlation were performed using SPSS (Statistical Package for the Social Sciences) Version 21.0. The values were represented in numbers (%) and mean ± SD. Inter-group comparisons were performed using the Pearson Chi-square test and when relevant, the Student’s t-test; P<0.0505 (95% confidence interval) was considered statistically significant.

## **RESULT**S

### Clinical Findings

The study included 84 cases, 77 patients with AML and seven with MDS. It also included 16 healthy controls. The patients' mean age (years) was 35.96±19.98. The male-to-female ratio was 1.7:1; however, on statistical comparisons, no significant difference was found between the two groups regarding age and gender. Fatigue was the most common symptom, followed by fever, weight loss, and bleeding manifestations.

### Hematological, morphological and epigenetic findings

#### Hematological findings

Statistically significant reduced hemoglobin (p<.001), platelet count (p<.001) and increased Total leukocyte count (p<.001) were found in cases as compared to the control group. The mean % of blasts in the peripheral blood smear was 42.98±31.77) and in the bone marrow aspirate smear was 57.54 ± 29.23 (**Table 2**).

**Table 2:** Clinical and Hematological characteristics of AML, MDS and controls included in the study

Variables (±SD)	Values	Cases (N=84)	Controls (N=16)	P value
Age (years)	35.96±19.98	36.27±20.36	34.33±18.41	<.679
Gender (M:F)		54:30	9:7	<.542
Symptoms and Signs				
• Fever	58	55	3	<0.0001
• Fatigue	76	73	3	<0.0001
• Bleeding manifestations	17	16	1	<0.270
• Weight loss	23	22	1	<0.082
• Hepatomegaly	27	27	0	-
• Splenomegaly	22	22	0	-
• Lymphadenopathy	9	9	0	-
Hematological parameters (Mean± SD)				
• Hemoglobin(gm/dl)		6.62±1.89	10.44±2.14	<0.001
• Total leukocyte counts (109/L)		34.29±51.48	11.45±44.07	<0.001
• Platelet counts (109/L)		67.67±84.46	260.62±135.66	<0.001
• Blasts % in peripheral smears		42.98±31.77	0.00	-
• Blasts% in Bone marrow		57.54±29.23	0.00	-

**Figure 1:**
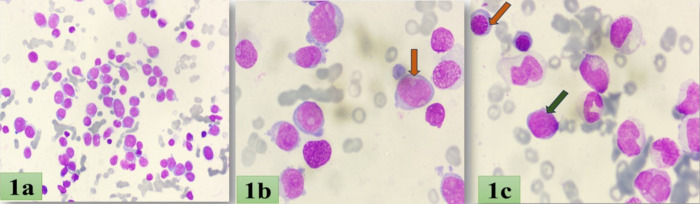
Photomicrographic findings in peripheral blood and bone marrow (a) Photomicrograph showing blast in peripheral blood smear; (b) Photomicrograph showing blast in bone marrow aspirate smear (Arrow showing prominent nucleoli); (c) Photomicrograph showing dysplastic changes in bone marrow aspirate smear (red arrow showing nucleated red cell; green arrow showing blast)

Morphologically, blasts were medium to large, having high nucleo-cytoplasmic ratio, coarse clumped to open chromatin, multiple prominent nucleoli and scant cytoplasm. Dysplastic changes were considered when > 10% of cells showed dysplastic changes in any lineages. Of 7 MDS patients, 2 were MDS with increased blast-1, 2 MDS with increased blast-2, 1 MDS with 5q deletion, and 2 MDS with low blast and ring sideroblasts (**Figure 1**).

#### Methylation findings

**Figure 2:**
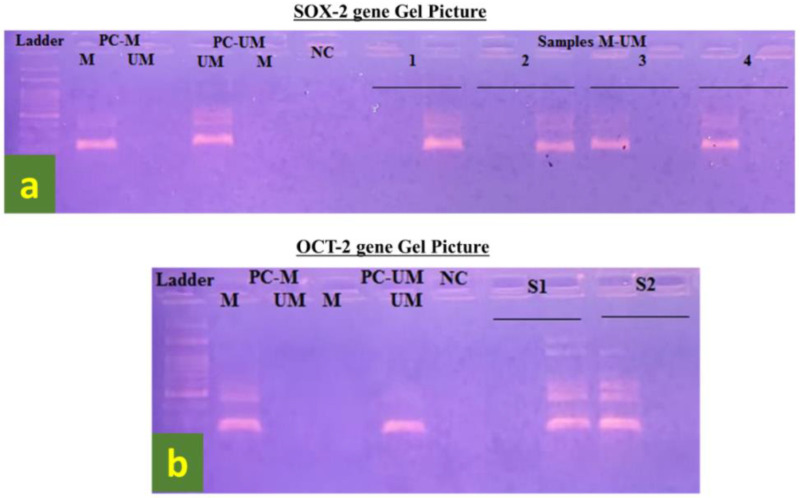
Methylation Status of SOX-2 and OCT-4 in AML Patients by Methylation-Specific PCR (MSP) (a) MSP analysis of SOX-2 in AML patients and standard control. PC: positive control for methylated and unmethylated; NC: negative control; Samples: 1,2,3,4; M: methylated; UM: unmethylated; (b) MSP analysis of OCT-4 in AML patients and standard control. PC: positive control for methylated and unmethylated; NC: negative control; Samples: 1 and 2; M: methylated; UM: unmethylated.

SOX-2 gene methylation was observed in a higher proportion of AML (n=48/77) and MDS (n=4/7) patients than in Controls (n=3/16) (**Figure 2**).

**Figure 3:**
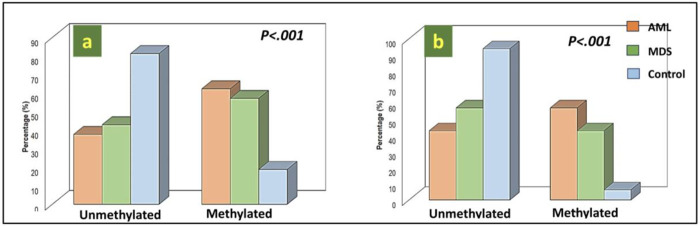
Comparative Methylation Profiles of SOX-2 and OCT-4 Across AML, MDS, and Control Groups (a) Histogram showing intergroup comparison between cases of AML, MDS, and controls of the SOX 2 gene displaying significant methylation (p<.001); (b) Histogram showing intergroup comparison between cases

On comparing statistically, this difference was significant (p<0.001). OCT-4 methylation was observed in a higher proportion of AML (n=44/77) as compared to MDS (n=3/7) and Controls (n=1/16). On comparing statistically, this difference was found to be significant (p<0.001) (**Figure 3**).

Only a tiny proportion of cases in the AML group had both SOX-2 and OCT-4 gene methylation (n=25/77) as compared to MDS (n=0/7) and Controls (n=0/16). On comparing statistically, this difference was also significant (p<0.007).

#### Methylation and dysplasia

Out of 77 AML cases, 54 showed no evidence of dysplasia, while 23 showed dysplasia in at least one lineage. SOX2 methylation was present in significant numbers in both cases without dysplasia (n=30/54) and with dysplasia (n=15/23). Regarding statistical comparison, this difference was not statistically significant (p>0.05).

OCT-4 methylation was present in many cases without dysplasia (n=27/54) but was lower in dysplasia (n=11/23). On comparing statistically, this difference was not significant (p>0.05). All MDS cases (n=7) showed dysplasia, but the instances enrolled were too few to draw any considerable conclusion of dysplasia and methylation of these two genes. Cytogenetic analysis was available for all 84 cases (n=77 AML and n=7 MDS). Of the 77 AML patients, normal cytogenetics were found in 46, and the remainder had cytogenetic abnormalities. The most common cytogenetic changes were t(8:21), followed by FLT3 mutation, Trisomy 8, t(15:17), and monosomy 5q deletion, SF3B1, 7q deletion, inv(3), t(6:9), and t(11q23) and complex karyotype. Of the 7 patients with MDS, 5 had normal cytogenetics, 1 had a 5q deletion, and 1 had an SF3B1 mutation. The correlation between cytogenetic abnormality and methylation status of SOX-2 and OCT-4 in each AML and MDS group was not statistically significant, as the numbers were too small in each subgroup to detect a correlation.

## DISCUSSION

Alterations in DNA methylation, including the hypomethylation of oncogenes and the hypermethylation of tumor suppressor genes, indicate that DNA methylation plays a vital role in leukemogenesis^25^. DNA methylation is the dominant mechanism for tumor suppressor gene (TSG) silencing and clonal variation in MDS evolution to AML^26^. Transcription factors such as OCT-4, SOX-2, C–myc, Klf-4, and Nanog are required for efficient self-renewal of normal hematopoietic stem cells.

Expression of these self-renewal regulatory factors like OCT-4, Nanog, SOX-2, nucleostemin, Zfx, Esrrb, Tcl1, Tbx 3, and Dppa4 have been studied in solid malignancies such as colon, prostate, and bladder carcinoma. However, little is known about their role in acute leukemia. The current study primarily focuses on aberrant methylation of 2 genes, SOX-2 and OCT-4, in AML and MDS patients. The majority of cases were young adults (61.9%) with a median age of 35 years (age range 19-60), followed by the pediatric (<18 years) population (23.8%), along with a small proportion of elderly patients (> 60 years) (14.3%). Male preponderance was observed (M: F ratio=1.7:1). Earlier study done by Zhang L Y, Yuan Y Q, et al. (2016)^25^ and Yin JY, Tang Q et al. (2015)^27^ observed the median age of patients, 63 and 60.5 years respectively with slight male preponderance (M: F=1.1:1 and 1.27:1). Gupta R, Rahman K, et al. (2017) observed that median age of presentation of MDS patients is decade younger in Indian scenario, correspond to findings in this study^28^.

Regarding epigenetic modifications, we found increased methylation of OCT-4 and SOX-2 genes in AML and MDS patients compared to normal healthy controls. Out of 77 AML patients, OCT-4 was methylated in 57.1%, while SOX-2 was methylated in 62.3% of cases. In 7 MDS cases, OCT-4 methylation was 42.9%, while SOX-2 was methylated in 57.1%. Together, both genes showed methylation in 32.5% of AML cases. The result was significant for SOX-2 methylation (p<0.006), OCT-4 methylation (p<0.001), and methylation of both genes together (p<0.007). Zhang LY, Yuan YQ, et al. (2016), in their study with 20 AML patients, reported a high level of global DNA methylation in the peripheral blood of acute myeloid leukemia patients (p<0.05). They also found that higher methylation status of OCT-4 and SOX-2 genes was associated with differential profile to demethylation therapy (p<0.029). A study by Xiang Y, Zhou X et al. (2018)^29^ with a sample size of 152 AML patients found an increased expression of OCT-4 mRNA in AML patients compared with the control (p<0.001). Similarly, Tosic N, Petrovic I, et al. (2018)^30^ reported seldom overexpression of SOX genes like SOX-2, SOX-3, SOX-11, SOX-14, SOX-18 genes in adult AML patients compared to standard bone marrow samples (p<0.023). They used 50 AML patients in their study. Figueroa ME, Skrabanek L et al. (2016)^31^ reported that MDS and secondary AML display marked aberrant promoter DNA hypermethylation in their research with 14 MDS patients. They also found that de novo AML displays a less extensive aberrant methylation pattern than secondary AML and MDS (**Table 3** and **Table 4**).

**Table 3:** Comparative overview of published studies reporting OCT-4 and SOX-2 methylation abnormalities in AML and MDS

Sn	Study	Diagnosis	Sample size(n)	Median age	OCT-4	SOX-2
1	^27^	AML	87	60.5	High expression of OCT-4	(P<0.096)
2	^25^	AML	20	63	Methylated (p<0.05)	Methylated (p<0.05)
3	^29^	AML	152	37	Increased OCT-4 mRNA expression (p<0.001)	Not done
4	^30^	AML	50	-	Not done	Overexpression (p<0.023)
5	^31^	MDS	14	-	Hypermethylation of promoter region genes (p<0.0005)	
6	^3^	AML and ALL	100	-		
7	This study	AML and MDS	84 AML=77 MDS=7	35	Methylated (p<0.001)	Methylated (p<0.006)

**Table 4:** Comparative overview of studies evaluating OCT-4 alterations and associated cytogenetic findings in AML

No.	Study	Sample size(n)	OCT-4	Cytogenetics
1	Xiang Y et al. 2018^29^	152	Higher expression	Complex karyotype p<0.001
2	This study	84	Methylated	Normal cytogenetics P<0.049

We compared the methylation of these two genes in different age groups of patients of AML and MDS (Paediatric, elderly, and other). Still, we could not find any significant correlation (p<0.776 for SOX-2 and p<0.656 for OCT-4). Zhang LY observed findings similar to those of Yuan YQ et al. (2016) in their study with 20 AML patients and a median age group of 63 years. They found no significant correlation between OCT-4, SOX-2 genes' methylation status, and different age groups and genders (p<0.56 for age and p<0.18 for gender). We also compared AML cases with dysplasia to the methylation status of OCT-4 and SOX-2 genes, but we were unable to find any significant correlation (p<0.241) for OCT-4 and p<0.280 for SOX-2). Xiang Y et al. found a significant correlation between OCT-4 expression and complex cytogenetics; however, they did not assess OCT -4 methylation status^29^. We did not find any significant correlation between methylation status of OCT-4 and cytogenetic abnormality in AML patients in our study.

It could be a convincing diagnostic and prognostic target in AML and MDS patients. Further understanding of the relationship among DNA methylation, genetic aberrations, and expression might provide insight into the pathogenesis of AML. Jian J (2023) provided a comprehensive evaluation of DNA methylation in AML and predicted it to be an independent prognostic risk factor^32^. Liu X (2026) found that the DNA methylation pattern during the course of treatment in AML with hypomethylating agents is a predictor of response^33^. Out of 84 cases of Acute leukemia and MDS, 36 patients expired within 18 months of survival follow-up. Thirty-six patients opted for chemotherapy, of which 22 completed induction, and 14 were in the maintenance phase. The remaining 12 were lost in follow-up due to the ongoing COVID-19 pandemic at the time of study. The study's primary limitations include the methylation of only two genes (SOX-2 and OCT-4), a single-center design, a lack of quantitative methylation assessment, an absence of gene-expression validation, a small control cohort, and a lack of longitudinal assessment after therapy. The smaller sample size in the MDS group limits generalizability within it.

## CONCLUSION

This study concluded that significant methylation of two promoter genes, OCT-4 and SOX-2, was observed in AML/MDS cases compared with controls. Together, both genes also showed considerable methylation. These findings highlight the potential role of methylation of stemness-associated transcription factors as a biomarker for disease characterization and identification of epigenetic therapeutic targets in AML and MDS. Since targeted therapies have changed the landscape of AML treatment, improving survival and quality of life, more needs to be done to help patients live better and longer. This study provides insight into the importance of genetic methylation in AML, which can further stratify the role of targeted therapy. The smaller sample size in the MDS group limits generalizability within MDS patients and warrants validation in larger studies.

## Highlights

**Current knowledge**

Aberrant DNA methylation contributes to leukemogenesis in AML and MDS, but the epigenetic regulation of stemness-associated transcription factors remains incompletely understood.

**Question**

Are SOX-2 and OCT-4 genes aberrantly methylated in AML and MDS, and do their methylation patterns differ from those of healthy controls?

**Discoveries**

SOX-2 and OCT-4 methylation is significantly increased in AML and MDS, especially concurrent methylation in AML, highlighting their potential as epigenetic biomarkers and therapeutic targets.

## Author Contributions

Conceptualization, GY, DS.; writing - original draft preparation GY, MK, DS.; writing - review and editing, GY, MK, DS.; figure preparation, DS, MK, SP.; project administration, RK, MJ, USS, SPV. All authors have read and agreed to the published version of the manuscript.

## Declaration of Generative AI and AI-assisted technologies

This work was done without the use of artificial intelligence (AI). No element of manuscript development involved AI.

## Publisher’s note

All claims expressed in this article are solely those of the authors and do not necessarily represent those of their affiliated organizations, or those of the publisher, the editors and the reviewers. Any product that may be evaluated in this article, or claim that may be made by its manufacturer, is not guaranteed or endorsed by the publisher.
